# Construction of a RFP-lacZα bicistronic reporter system and its application in lead biosensing

**DOI:** 10.1371/journal.pone.0228456

**Published:** 2020-01-30

**Authors:** Chang-ye Hui, Yan Guo, Lisa Liu, Hao-qu Zheng, Chao-xian Gao, Wen Zhang

**Affiliations:** 1 Department of Pathology & Toxicology, Shenzhen Prevention and Treatment Center for Occupational Diseases, Shenzhen, China; 2 Department of Science & Education, Shenzhen Prevention and Treatment Center for Occupational Diseases, Shenzhen, China; 3 Institute of Translational Medicine, Shenzhen Second People’s Hospital, Shenzhen, China; Instituto Butantan, BRAZIL

## Abstract

The combination of a fluorescent reporter and enzymatic reporter provides a flexible and versatile way for the study of diverse biological processes, such as the detection of transcription and translation. Thus, there is an urgent need to develop this novel bifunctional reporter system. This study reports the design, construction, and validation of a new dicistronic mCherry-lacZα reporter system by artificial *lac* operon and *pbr* operon models in lacZM15-producing *E*. *coli*. It allows two reporter genes to be co-transcribed into a dicistronic mRNA strand, followed by coupled expression of mCherry and lacZα. In artificial *lac* operons, expression of the downstream lacZα was demonstrated to be positively related to expression of the upstream ORF. In artificial *pbr* operons, compared with the insertion of downstream full-length lacZ, the insertion of downstream lacZα exerted a slight effect on the response from the upstream mCherry. Furthermore, the downstream lacZα reporter showed stronger response to Pb(II) than the downstream full-length lacZ. Importantly, the response sensitivity of downstream lacZα was still higher than that of upstream mCherry in a dual RFP-lacZα reporter construct. The highly efficient expression profile of the reporter lacZα peptide makes it a preferred downstream reporter in polycistronic constructs. This novel bifunctional reporter system offers a robust tool for biological studies.

## Introduction

Recombinant production of enzymatic and fluorescent reporters has greatly improved our ability to explore the potential molecular processes underlying gene expression and regulation in various living organisms [1, 2]. In particular, the fluorescent proteins have been widely used to study promoter activity and regulation, protein function and localization, and non-invasive imaging over many years [3, 4]. Since their discovery, fluorescent proteins have been extensively modified to improve stability, brightness, and oligomerization state [5]. Reporter enzymes, such as β-galactosidase, hydrolyze externally supplied substrates, and produce detectable signals. They have been widely used for promoter identification and gene expression analysis in many organisms [6, 7]. Compared with fluorescent reporters, enzymatic reporters have an enhanced sensitivity with low background reactivity.

The widely used fluorescent and enzymatic reporters also exhibit strengths and limitations. Fluorescent reporters can be directly detected without the supplement of external substrates, and thus many available single-cell methodologies are based on fluorescent reporters [1, 8]. However, a fluorescent protein molecule has only one chromophore, and photobleaching of the fluorophore is inevitable. As a result, fluorescence signal is difficult to be detected when the expression is weak, especially in culture media or in living organisms with high autofluorescence background [5, 9]. The greatest advantage of enzymatic methods is its high sensitivity, because the detectable products hydrolyzed by a low-level enzyme can be accumulated until they can be detected [10]. In addition, a convenient plate assay can be used for naked-eye colorimetric detection of enzymatic activity [6, 11]. Although enzymatic reporter assays usually provide valuable information concerning the regulation of gene expression within a population [6], a high-throughput method for single-cell analysis based on a fluorogenic substrate was also successfully developed. Quantitative measurements of near-basal expression of β-galactosidase in single bacterial cells were demonstrated [12].

To combine the advantages of enzymatic and fluorescent reporters, many attempts have been made to develop versatile reporter systems in recent years. For example, the development of hybrid bifunctional reporter proteins [7, 9, 13], dual promoter-driven reporter systems [14], and even the combination of two reporters in a single dicistronic unit [6, 15, 16]. The expression of the reporter is affected by many factors, including molecular weight of the product, codon preference, secondary structure of the mRNA, strength of the promoter, RBS variants with different translational efficiencies, and more [17–19]. Previous studies have demonstrated that the co-expression of several genes could be regulated in an artificial operon by assembling them in tandem, but the expression of the upstream gene was always higher than that of the downstream one [17]. The molecular sizes of commonly used reporters, such as fluorescence protein, luciferase, and β-galactosidase, range from several hundred to more than a thousand amino acids [2]. Unfortunately, it is difficult to detect weak transcriptional signals from the target promoter based on co-expression of several large molecular weight reporters in the host [15, 20]. Protein expression is regulated precisely by changing the combination of genetic codes in engineered bacterial cells [21]. The translation efficiency of the upstream open reading frame (ORF) could also be easily adjusted using the inserted rare codons. Thus, the expression of the downstream ORF in the polycistronic unit can be further decreased [22, 23].

The development of dual reporter systems based on two different detection principles facilitated the characterization of transcriptional and translational levels of the target region. However, the responses from both reporters were not synchronized, and a high level of expression of the upstream gene was seen in prokaryotes in most situations [6, 24–26]. It is well understood that the production of a smaller reporter is always more efficient and energy-saving for microbial cells [17]. The truncated reporter peptide lacZα, a short peptide of 56 amino acids, was previously developed for improved monitoring of low-level transcription in lacZM15-producing bacteria [11]. Compared with the full-length β-galactosidase, truncated polypeptide lacZα expression decreases energy consumption of the host cells, and thus enhanced lacZα expression and detection sensitivity are expected in a dicistronic reporter system with lacZα as the downstream reporter. To improve the expression level of a downstream reporter in a dicistronic molecular device, the lacZα peptide should be a preferred alternative to traditional high molecular weight reporters, such as the full-length β-galactosidase.

In this paper, we have developed a novel RFP-lacZα reporter cassette, which is based on a dicistronic genetic structure, and allows for the simultaneous expression of two reporter genes in a regulated fashion. This cassette is designed to allow for the insertion of a target promoter in front of it in order to construct a RFP-lacZα reporter system. Although the expression of the upstream reporter mCherry was greatly decreased by inserting consecutive rare codons, the expression of the downstream reporter lacZα was only slightly affected. More importantly, the detection sensitivity of downstream lacZα was always higher than that of upstream mCherry in an artificial *lac* operon. This RFP-lacZα reporter system has been instrumental for Pb(II) biosensing based on an artificial *pbr* operon. An engineered lead biosensor with two different reporters in a single genetic construct responded to low levels of lead by simultaneously generating fluorescent signal and β-galactosidase activity. The characteristics of a flexible lead biosensor allows for an expanded application range.

## Materials and methods

### Bacterial strains, plasmids, and agents

The bacterial strains and vectors involved in this study are listed in Table 1. *E*. *coli* Top 10 was used as a host for the cloning and induced expression of the plasmid-coded reporter proteins. *E*. *coli* was grown in Luria Broth (OXOID, Basingstoke, UK) at 37°C. Pyrobest DNA polymerase, restriction enzymes, and gel extraction kits were purchased from TaKaRa (Dalian, China). Isopropyl-β-D-thiogalactopyranoside (IPTG), o-nitrophenyl-β-D-galactopyranoside (ONPG), 5-bromo-4-chloro-3-indolyl-β-D-galactoside (X-gal), ampicillin, and lysozyme were obtained from Sangon Biotech (Shanghai, China). All chemicals were purchased from Sigma-Aldrich (St Louis, MO, USA). All oligonucleotides were synthesized by Sangon Biotech (Shanghai, China).

**Table 1:** Bacterial strain, plasmids, and primers used in this study.

Strain and vectors	Genotypes or description	Reference
Strain		
*E*. *coli* Top10	F^-^, Φ80*lac*ZΔM15, Δ*lac*X74, *rec*A1	Invitrogen
Plasmid		
pBR322	Amp^R^, Tet^R^, *ori pMB1*, commonly used *E*. *coli* cloning vector	Novagen
pT-RFP	T vector carrying *mCherry*	[22]
pPlac-lacZα	Amp^R^, *ori pMB1*, pBR322 derivative with lacZα peptide expressing under *lac* promoter	[11]
pPlac-ORF-lacZα	pPlac-lacZα derivative with insertion of MCS-containing ORF	This study
pPlac-mu1ORF-lacZα	pPlac-ORF-lacZα derivative with one rare codon in ORF	This study
pPlac-mu2ORF-lacZα	pPlac-ORF-lacZα derivative with two consecutive rare codons in ORF	This study
pPlac-mu3ORF-lacZα	pPlac-ORF-lacZα derivative with three consecutive rare codons in ORF	This study
pPlac-RFP-lacZα	pPlac-ORF-lacZα derivative with *mCherry* in ORF, a dual RFP- lacZα reporter system	This study
pPlac-mu2RFP-lacZα	pPlac-RFP-lacZα derivative with two consecutive rare codons preceding *mCherry*	This study
pPlac-RFPmu3-lacZα	pPlac-RFP-lacZα derivative with three consecutive rare codons following *mCherry*	This study
pT-Ppbr	pUCm-T carrying *pbrR* and divergent *pbr* promoter region	[11]
pPpbr-RFP	pBR322 derivative with *pbrR* and *pbr* promoter preceding *mCherry*, a single RFP reporter system	[11]
pPpbr-RFP-lacZα	pPlac-RFP-lacZα derivative with *pbrR* and *pbr* promoter preceding *mCherry* and *lacZα*, a dual RFP-lacZα reporter system	This study
pPlac-lacZ	pBR322 derivative with β-galactosidase expressing under lac promoter	[11]
pPpbr-RFP-lacZ	pPpbr-RFP-lacZα derivative with *pbrR* and *pbr* promoter preceding *mCherry* and full-length *lacZ*, a dual RFP-lacZ reporter system	This study

### Construction of a series of dicistronic plasmids

All the primers involved in this study are listed in S1 Table, and PCR reactions were optimized for each primer pair. Plasmid pPlac-lacZα containing the *Plac*-*lacZα*-*rrnB* cassette was previously constructed to facilitate validation of a monocistronic lacZα reporter [11]. The pPlac-ORF-lacZα was constructed by the overlapping extension PCR method. The *Plac*-*ORF* cassette containing a new multiple cloning site (MCS) was amplified from pPlac-lacZα using primers F-Plac and R-Plac-ORF. The *ORF*-*lacZα*-*rrn*B terminator cassette was amplified from pPlac-lacZα using primers F-ORF-lacZα and R-Ter. The amplified products from both of the above reactions were added to another PCR reaction using primers F-Plac and R-Ter. The resulting *Plac*-*ORF*-*lacZα*-*rrn*B terminator gene fusion cassette was cloned as a *Nde*I/*Eco*RI fragment into pBR322 to create the plasmid pPlac-ORF-lacZα. DNA fragments were amplified from pPlac-ORF-lacZα using primers R-Ter and F-mu1ORF, F-mu2ORF, or F-mu3ORF, then digested and inserted into pPlac-ORF-lacZα via *Hin*dIII and *Eco*RI sites, generating pPlac-mu1ORF-lacZα, pPlac-mu2ORF-lacZα, and pPlac-mu3ORF-lacZα, respectively.

To assemble a dual reporter system, the gene of mCherry was amplified from pT-RFP using primers F-RFP and R-RFP, and this fragment was then cloned as a *Hin*dIII and *Xho*I fragment into pPlac-ORF-lacZα, previously digested with the same enzymes. The resulting recombinant plasmid was named pPlac-RFP-lacZα with a dual RFP-lacZα reporter cassette in it. A fragment with two consecutive rare codons preceding *mCherry* was amplified from pPlac-RFP-lacZα by PCR with primers F-mu2RFP and R-RFP, and inserted into the *Hin*dIII and *Xho*I sites of pPlac-RFP-lacZα to generate pPlac-mu2RFP-lacZα. Likewise, a fragment with three consecutive rare codons following *mCherry* was amplified from pPlac-RFP-lacZα by PCR with primers F-RFP and R-RFPmu3, and inserted into the *Hin*dIII and *Xho*I sites of pPlac-RFP-lacZα to generate pPlac-RFPmu3-lacZα.

**Fig 1:**
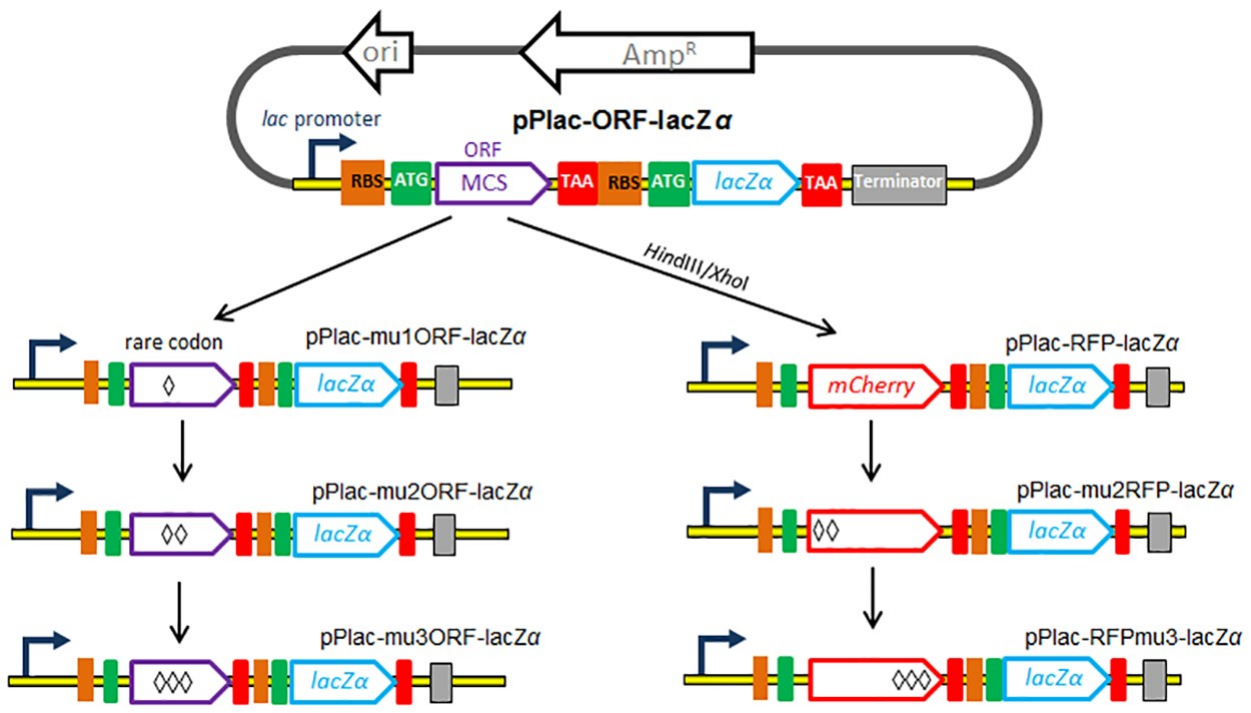
Design and assembly of the dual reporter constructs and their functional characterization. Schematic representation of the main features of a series of *lac* promoter reporter vectors, depicting the two reporters mCherry and lacZα separated by a stop codon and an extra RBS, under the control of a *lac* promoter.

The strategy used for assessing *lac* promoter activity using various reporter cassettes is summarized in Fig 1, and the cloning and expression regions of recombinant plasmids involved in the study are shown in S1 Fig. A 2063 bp *Nde*I-*Eco*RI fragment derived from pBR322, which only retains the ampicillin resistance cassette and the origin of replication, was chosen as the clone vector backbone. A DNA fragment containing extra RBS and an ORF was first inserted into pPlac-lacZα after the *lac* promoter region to generate pPlac-ORF-lacZα. As shown in S2A Fig, a short upstream ORF composed of a multiple clone site (MCS) and a His Tag, together encoding a 14-amino acid peptide (amino acid sequence: MKLGSLEHHHHHH), was inserted into this dicistronic plasmid. One, two, and three consecutive rare codons were then introduced following the *Hin*dIII site in the short ORF, generating pPlac-mu1ORF-lacZα, pPlac-mu2ORF-lacZα, and pPlac-mu3ORF-lacZα, respectively. The mCherry coding sequence was inserted into the short ORF to generate a dual mCherry-lacZα reporter plasmid pPlac-RFP-lacZα, as shown in S2B Fig. Two consecutive rare codons were then placed upstream of *mCherry*, and three consecutive rare codons were placed downstream of *mCherry*, to generate pPlac-mu2RFP-lacZα and pPlac-RFPmu3-lacZα, respectively.

### Assembly of the plasmid-based lead biosensor

A dual mCherry-lacZα reporter element was amplified from pPlac-RFP-lacZα, and fused to the genetic element containing *pbrR* and divergent *pbr* promoter by an overlap extension PCR method. In brief, a dual mCherry-lacZα reporter element was amplified with the primer pair F-Ppbr-RFP and R-Ter. The cassette containing *pbrR* and *pbr* promoter was amplified from pT-Ppbr using the primer pair F-Ppbr and R-Ppbr-RFP. Both of the amplified fragments from pPlac-RFP-lacZα and pT-Ppbr were added to another PCR reaction using primers F-Ppbr and R-Ter to generate the *pbrR*-*Ppbr*-*mCherry*-*lacZα* gene fusion cassette. The amplified fragment and pBR322 were then digested with *Nde*I and *Eco*RI, and ligated together to create pPpbr-RFP-lacZα.

Furthermore, a dual mCherry-lacZ reporter cassette was also fused with *pbrR-Ppbr* to assemble pPpbr-RFP-lacZ by an overlap extension PCR. Briefly, the *pbrR-Ppbr*-*mCherry* cassette was first amplified from pPpbr-RFP-lacZα using primers F-Ppbr and R-Ppbr-RFP-lacZ. The *lacZ*-*rrn*B terminator cassette was then amplified from pPlac-lacZ using primers F-Ppbr-RFP-lacZ and R-Ter. Both of the amplified fragments were finally mixed to be amplified with primers F-Ppbr and R-Ter to generate the *pbrR*-*Ppbr*-*mCherry*-*lacZ* gene fusion cassette, which was inserted into the *Nde*I and *Eco*RI sites of pBR322 to generate pPpbr-RFP-lacZ.

### Reporter genes expressions

Standard procedures were employed for the transformation of *E*. *coli* Top10 [27]. The transformed *E*. *coli* cells were spread on LB agar plates containing 50 μg/mL ampicillin, and then cultured overnight at 37°C. A single colony was used to inoculate LB medium supplemented with 50 μg/mL ampicillin, and was cultured at 37°C for 12 h. The precultured recombinant *E*. *coli* culture was diluted to an OD_600_ of 0.01 in fresh LB medium containing 50 μg/mL ampicillin, and grown to an optical density of 0.4 at 600 nm. For the reporter genes expression under the *lac* promoter, the cultures were then induced with 0–1.0 mM IPTG. For the reporter genes expression under the *pbr* promoter, the cultures were then induced with varying concentrations of Pb(II) plus 0.1 mM IPTG before assessing functional lacZα and mCherry expression.

### β-galactosidase assay

The β-galactosidase activity of the induced culture was measured as described by Miller with ONPG as the substrate [28]. A semi-quantitative detection method to study β-galactosidase activity was developed in this study. In brief, blot paper was divided into 1*1 cm squares and immersed in lysis buffer (50 mM PBS, 200 mM NaCl, 1 g/L lysozyme, and 2 mM X-gal,). After the excess lysis buffer was removed, an aliquot of 10 μL of induced culture was dripped onto the blot paper, and incubated at 37°C for 15 min before drying at 50°C.

### mCherry assay

The fluorescent signal of mCherry produced in recombinant *E*. *coli* was measured with the Lumina fluorescence spectrometer (Thermo, USA) as previously described [22]. Fluorescence emission was recorded at 610 nm for mCherry, and the fluorescence value was normalized by dividing the fluorescence intensity by the OD_600_ value of the same sample. A dot blot using cell lysate was performed as previously described [29]. Briefly, twenty microliters of induced culture was mixed with an equal volume of assay solution (200 mM Tris-HCl, pH 6.8, 2% SDS, and 200 mM DTT), and boiled for 10 min. An aliquot of 5 μL of each sample was dripped onto PVDF membrane. After the membrane was blocked and washed, it was incubated with primary mouse anti-His IgG (Tiangen, Beijing, China). After extensive washing, the membrane was then incubated with a secondary HRP labeled rabbit anti-mouse IgG (Sangon Biotech, Shanghai, China). Finally, the associated antibodies were visualized using an enhanced HRP-DAB chromogenic substrate kit (Tiangen, Beijing, China).

## Results and discussion

### Relationship between lacZα expression in the downstream ORF and rare codons in the upstream ORF

**Fig 2:**
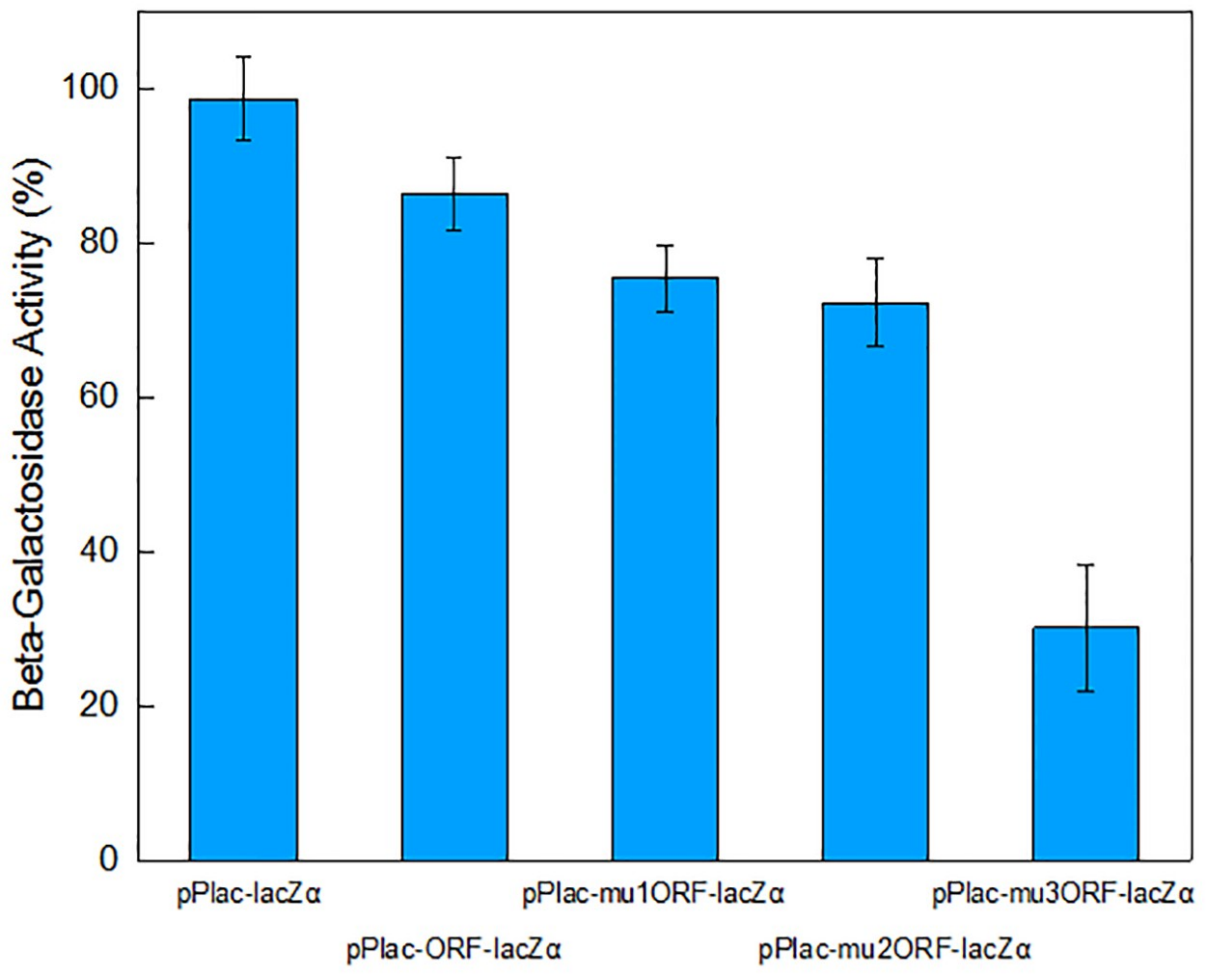
Effect of rare codons in an upstream ORF on the response from a downstream lacZα reporter system. Overnight cultures were inoculated into fresh LB media, then the cells harboring various vectors were exposed to 1.0 mM IPTG at the logarithmic growth phase. After 4 h incubation at 37°C, the β-galactosidase activities were determined. The β-galactosidase activity is shown as percentage of β-galactosidase activity in Top10/pPlac-lacZα, which is defined as 100%. Levels of β-galactosidase activity are expressed as mean ± SD, n = 3.

Several dicistronic reporter systems have been successfully constructed using synthetic biological methods. The expression of α-donor lacZα peptide is driven by the detected promoter in the target vector, and IPTG-inducible expression of α-acceptor lacZM15 is encoded in the host genome. Both of them can be assembled by α-complementation to become an active enzyme in the host cell. Then, the routine β-galactosidase assay can be performed. We next determined the function of these reporters in lacZM15-producing *E*. *coli* Top10. To validate the potential of lacZα as the downstream reporter, an extra ORF was first placed upstream of the lacZα reporter cassette to construct a dicistronic vector pPlac-ORF-lacZα. The upstream ORF is composed of a MCS used for the insertion of another reporter gene and a His Tag used for the blot assays. The upstream short ORF encodes a single 14-amino acid peptide. The strength of RBS, mRNA stability, and the percent of rare codons have been previously demonstrated to affect the translational efficiency of the downstream genes [4, 17]. In the study, one, two, and three consecutive rare codons were introduced into the upstream ORF to facilitate the detection of response change from the downstream lacZα reporter system. The *lac* promoter activity was then evaluated by the measurement of downstream lacZα production-inducing β-galactosidase activity, and the results are shown in Fig 2.

As expected, the highest enzymatic response came from the lacZα monocistronic reporter system Top10/pPlac-lacZα. The introduction of a short upstream ORF decreased the response from downstream lacZα, and the rare codons introduced into the upstream ORF resulted in the further decrease of the lacZα response. Three rare codons chosen in this study (CTA, ATA and AGG) were used at a frequency of < 0.5% in *E*. *coli* [30], and our previous studies have demonstrated that three consecutive rare codons in upstream ORF could cause expression failure of downstream mCherry [22]. Interestingly, even though three consecutive rare codons were introduced into the upstream ORF, a strong enough lacZα response was still detected in Top10/pPlac-mu3ORF-lacZα.

Translational coupling is the interdependence of expression of multiple neighboring genes encoded in an artificial operon. The coupling mainly depends on the distance between the stop codon of the upstream ORF and the start codon of the downstream ORF [4, 31]. Our studies suggest that the development of a downstream reporter that is easily and efficiently expressed in the host, such as a lacZα peptide, could be another preferred option for the improvement of translational coupling in the dual reporter system. Previously intractable problems, such as a response delay from the downstream reporter [22] or a decreased detection sensitivity of the downstream reporter [6, 16], may be resolved through using this modified system.

### Comparison of upstream mCherry and downstream lacZα reporter for *lac* promoter activity

**Fig 3:**
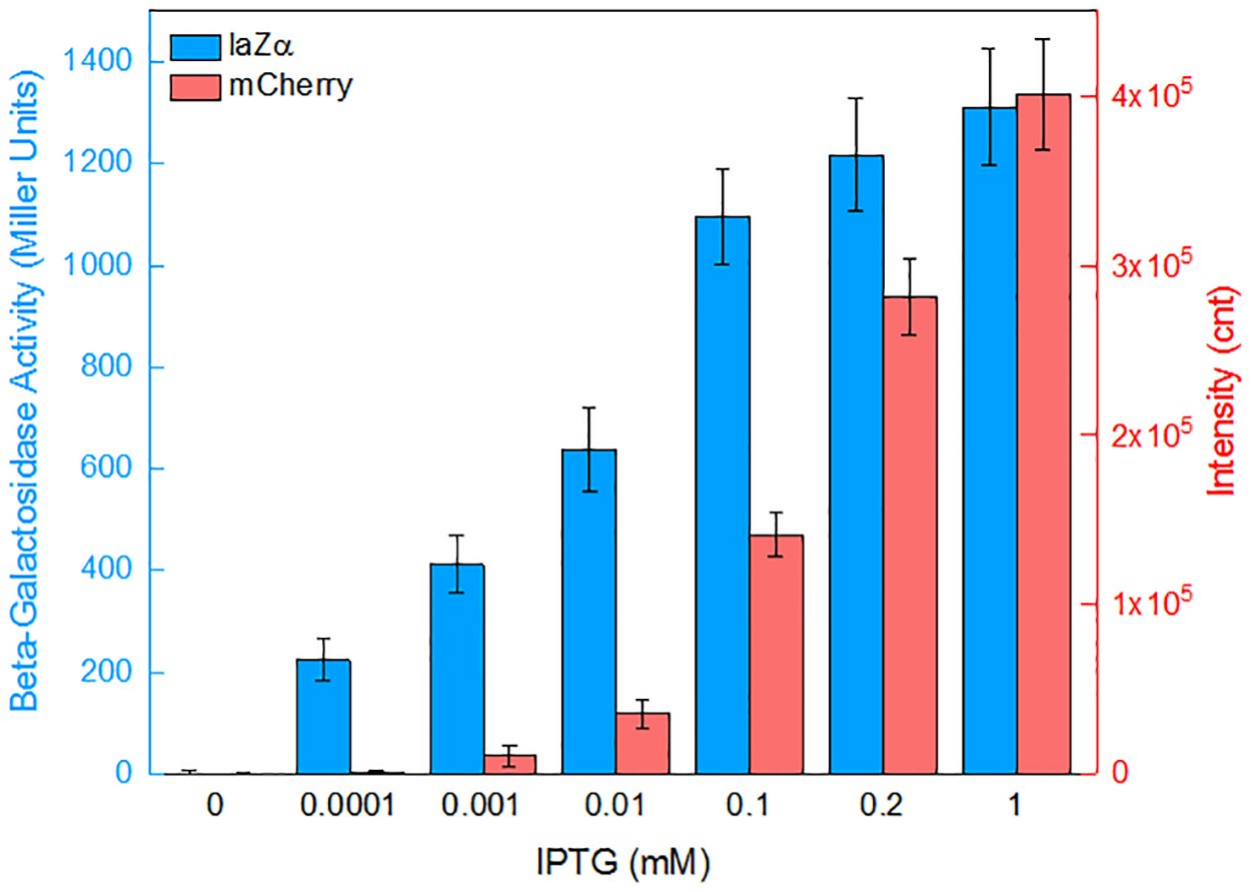
Response of Top10/pPlac-RFP-lacZα to different concentrations of IPTG. Recombinant *E*. *coli* was induced with 0–1 mM IPTG at 37 °C for 6 h. Then, the β-galactosidase activity and mCherry fluorescent signal of the induced cultures were determined.

To further validate the advantage of lacZα as the downstream reporter of a dual reporter system, the mCherry gene was firstly inserted into the upstream ORF to assemble a dicistronic reporter construct pPlac-RFP-lacZα. As shown in Fig 3, reports of both mCherry and lacZα were positively related to the concentration of inducer IPTG. Upstream mCherry was found to respond to 0.001 mM IPTG. However, even when the concentration of IPTG was as low as 0.0001 mM, strong enough β-galactosidase activity was still detected. Due to the highly efficient expression of a lacZα peptide, a similar or reduced detection limit may be expected when lacZα is chosen as a downstream reporter in a dicistronic construct.

To test whether or not the red fluorescent signal from the upstream ORF is positively related to the downstream lacZα production-inducing β-galactosidase activity, two and three consecutive rare codons were then introduced into the mCherry ORF.

**Fig 4:**
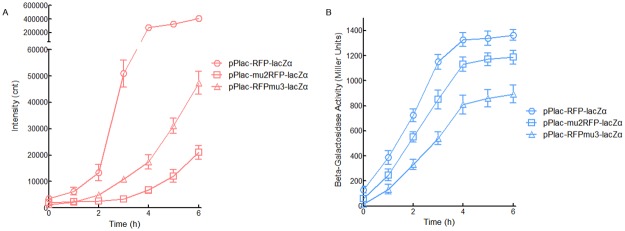
Validation of the dual mCherry-lacZα reporter system. Top10/pPlac-RFP-lacZα, Top10/pPlac-mu2RFP-lacZα, and Top10/pPlac-RFPmu3-lacZα were exposed to 1.0 mM IPTG at the logarithmic growth phase. The mCherry fluorescence (A) and β-galactosidase activity (B) were determined at different time intervals after 1.0 mM IPTG induction. Data are mean ± SD from three independent assays each from three independent cultivations.

The *lac* promoter activity was evaluated by assays for both mCherry and β-galactosidase activity. Rare codons located in upstream *mCherry* greatly down regulated the expression of mCherry. However, the production of downstream lacZα was slightly decreased (S3 Fig). This is different from the performance of traditional reporters, and a previous study has shown that rare codons located in the upstream ORF dramatically decreased the expression of downstream fluorescent protein in a dicistronic unit [22]. Lactose analogue-inducible promoters exhibit leaky expression in LB medium [32]. Thus, the expression of mCherry and lacZα were detected at 0 h in a blot dot test (S4A Fig), and a semi-quantitative paper test (S4B Fig), respectively. The highest responses of both mCherry and lacZα all came from Top10/pPlac-RFP-lacZα in all tests. Although two rare codons located in the 5’-terminal region of *mCherry* exerted a stronger negative influence on the yield of mCherry than that of the three rare codons located in its 3’-terminal region (Fig 4A, S4A Fig), the decrease in the response from the downstream lacZα was still positively related to the amount of rare codons located in upstream *mCherry* (Fig 4B, S4B Fig). It is worth mentioning that three consecutive rare codons located in the mCherry ORF could not block the response from the downstream lacZα (Fig 4B, S4B Fig). These results demonstrate the power of the lacZα reporter as a highly sensitive candidate for the downstream reporter to assess transcriptional activity of the target promoter in a dicistronic reporter construct.

### Validation of the dual RFP-lacZα reporter in lead biosensing

**Fig 5:**
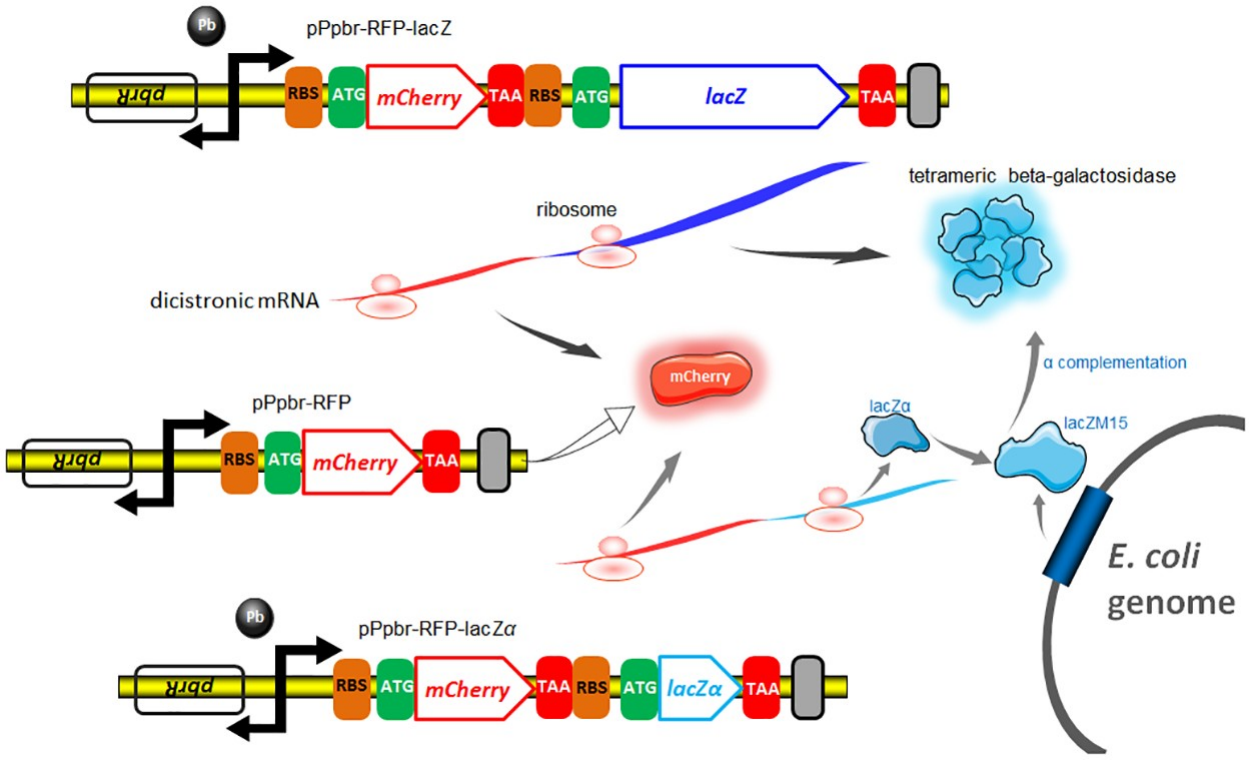
Genetic organization of three different lead biosensors. Based on a dicistronic pPpbr-RFP-lacZ, both mCherry and active β-galactosidase are expressed with Pb(II) induction. Based on a monocistronic pPpbr-RFP, only the mCherry reporter is expressed with Pb(II) induction. Based on a dicistronic pPpbr-RFP-lacZα, the mCherry reporter and lacZα are expressed under the control of a target *pbr* promoter driven by Pb(II), and lacZM15 is synthesized under the control of a host *lac* promoter driven by IPTG. After active tetrameric β-galactosidase is finally assembled in the host cell, both fluorescent signal and enzymatic activity are then determined.

Based on the natural *pbr* operon originating from *Cupriavidus metallidurans* CH34, several lead bacterial biosensors based on a monocistronic construct have been successfully developed, and fluorescence proteins were all chosen as reporters [11, 33, 34]. The *lac* promoter region (171 bp) in pPlac-RFP-lacZα, was substituted with a cassette containing the *pbrR* gene (435 bp) and divergent *pbr* promoter region (85 bp) to generate pPpbr-RFP-lacZα. To compare the reporter level of a lacZα peptide with that of the full-length β-galactosidase, a dicistronic pPpbr-RFP-lacZ was constructed, and traditional monocistronic pPpbr-RFP was also involved in the study. The genetic elements of whole cell lead biosensors and the potential molecular mechanism involved in a single mCherry reporter system, dual mCherry-lacZα reporter system, and a dual mCherry-lacZ reporter system are shown in Fig 5.

**Fig 6:**
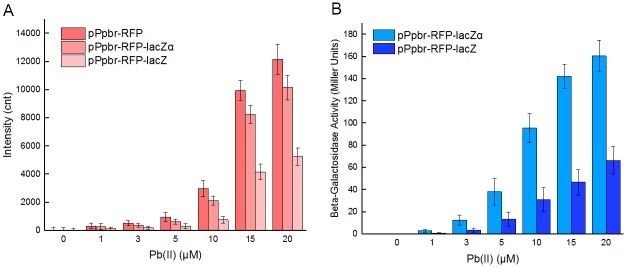
Monocistronic RFP, bicistronic RFP-lacZα, and bicistronic RFP-lacZ reporter strains in response to lead(II). The fluorescent (A) and enzymatic (B) response of Top10/pPpbr-RFP, pPpbr-RFP-lacZα, and pPpbr-RFP-lacZ to different concentrations of Pb(II) plus 0.1 mM IPTG after 4 h incubation at 37°C. Data are mean ± SD from three independent experiments each from three independent cultivations.

Fluorescent signals generated from all three constructs are shown in Fig 6A. Compared with monocistroinc pPpbr-RFP, the fluorescent signal from the dicistronic pPpbr-RFP-lacZα was slightly decreased, and both of them responded to Pb(II) at concentrations as low as 10 μM. However, the fluorescent signal from dicistronic pPpbr-RFP-lacZ was greatly decreased, and only responded to Pb(II) at 15 μM. Enzymatic signals generated from both dicistronic constructs are shown in Fig 6B. Both of them were positively related to the concentration of Pb(II), and responded to Pb(II) at concentrations as low as 3 μM. Interestingly, enzymatic activity derived from the downstream lacZα was always significantly higher than that derived from the downstream full-length lacZ. In summary, the upstream mCherry response to Pb(II) was slightly affected by the introduction of downstream lacZα in this pPpbr-RFP-lacZα construct, a lead dual reporter biosensor. More importantly, the downstream lacZα response to Pb(II) was still more sensitive than the upstream mCherry.

**Fig 7:**
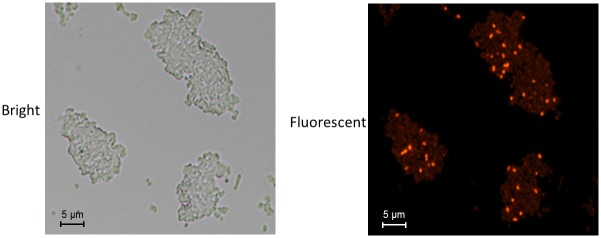
Fluorescence microscopic analysis of Top10/pPpbr-RFP-lacZα bacterial lawn picked from the LB agar plate treated with 20 μM Pb(II) plus 0.1 mM IPTG. Bacterial cells were visualized on slides using a Nikon Eclipse Ni fluorescence microscope (X400 magnification).

Both the accumulation of mCherry and the activation of β-galactosidase could also be clearly observed by the naked eye in a plate assay (S5 Fig). In addition, the implementation of a dual reporter system allows for Pb(II) biosensing at both the population and single-cell levels. The upstream mCherry reporter enabled easy quantification of response strength in single Pb(II) biosensor cells via fluorescent signals. Vibrant bacterial biosensor cells responded to Pb(II) strongly, and emitted bright red fluorescence. These cells could be easily distinguished under a fluorescence microscope with 400X magnification (Fig 7).

## Conclusions

In the current study, we describe the implementation and validation of a dual dicistronic reporter system based on the mCherry protein and a lacZα peptide. The successful production of both mCherry fluorescence and lacZα-inducing β-galactosidase activity in a series of artificial *lac* operons validated the functionality of this system. Due to the highly efficient expression of the lacZα peptide, the response strength of downstream lacZα was significantly higher that of downstream full-length lacZ. More importantly, the response sensitivity of downstream lacZα was even higher than that of upstream mCherry in a dual reporter system for Pb(II) biosensing. It is anticipated that the dual mCherry-lacZα reporter system, as a versatile bifunctional reporter system, will be useful in many future biological studies.

## Supporting information

S1 Table Primers used in this study. (XLSX)

S1 Fig The cloning/expression region of recombinant plasmids used in this study. DNA sequence and annotation data are all marked. (A) The cloning/expression region of pPlac-ORF-lacZα. Another open reading frame (ORF) containing multiple cloning sites (MCS) is inserted immediately preceding the expression region of lacZα. (B) The cloning/expression region of pPlac-mu1ORF-lacZα, pPlac-mu2ORF-lacZα, and pPlac-mu3ORF-lacZα. One rare codon, two consecutive codons, or three consecutive codons were introduced into the first ORF in each pPlac-ORF-lacZα, respectively. (C) The cloning/expression region of the dicistronic expression vector pPlac-RFP-lacZα. The mCherry coding sequence was inserted as a *Hind*III/*Xho*l fragment into pPlac-ORF-lacZα. (D) The cloning/expression region of pPlac-mu2RFP-lacZα and pPlac-RFPmu3-lacZα. Two consecutive rare codons were inserted in front of *mCherry* to generate pPlac-mu2RFP-lacZα, and three consecutive rare codons were inserted behind *mCherry* to generate pPlac-RFPmu3-lacZα. (E) The cloning/expression region of pPpbr-RFP. The cassette including the *pbrR* gene and the divergent *pbr* promoter was inserted in front of *mCherry*. (F) The cloning/expression region of pPpbr-RFP-lacZα. The cassette including the *pbrR* gene and the divergent *pbr* promoter was inserted in front of the dicistronic mCherry-lacZα genetic element. (G) The cloning/expression region of pPpbr-RFP-lacZ. The cassette including the *pbrR* gene and the divergent *pbr* promoter was inserted in front of the dicistronic mCherry-lacZ genetic element.
(TIFF)

S2 Fig *lac* promoter dicistronic reporter constructs with rare codons inserted into the upstream ORF. (A) The structure and DNA sequence of the ORF-lacZα reporter cassette in pPlac-ORF-lacZα. One, two, and three consecutive rare codons were introduced into the upstream ORF of pPlac-ORF-lacα after the *Hin*dIII site, generating pPlac-mu1ORF-lacZα, pPlac-mu2ORF-lacZα, and pPlac-mu3ORF-lacZα, respectively. (B) The structure and DNA sequence of the dual mCherry-lacZα reporter cassette in pPlac-RFP-lacZα, pPlac-mu2RFP-lacZα (containing two consecutive rare codons preceding *mCherry* gene), and pPlac-RFPmu3-lacZα (containing three consecutive rare codons following *mCherry* gene).
(TIF)

S3 Fig Comparison of the co-expression levels of mCherry and lacZα in three recombinant *E*. *coli* at 6 h after 1.0 mM IPTG induction. The mCherry fluorescence and β-galactosidase activity are shown as a percentage of the corresponding values in Top10/pPlac-RFP-lacZα, which are defined as 100%. The values are expressed as mean ± SD, n = 3.
(TIF)

S4 Fig Assay of two reporters from three *lac* promoter reporter dicistronic constructs. To assess mCherry production and β-galactosidase activity semi-quantitatively, a dot blot assay (A) was done to detect the His tag fused to the C-terminus of recombinant mCherry at different time intervals. A fast blot paper-based chromogenic method (B) was set up, and X-gal was chosen to be the substrate.
(TIF)

S5 Fig Co-occurrence of mCherry and β-galactosidase activity in plate assay with exposure to 20 μM Pb(II) plus 0.1 mM IPTG. Recombinant Top10/pPpbr-RFP-lacZα was streaked on a LB agar plate containing 20 μM Pb(II), 0.1 mM IPTG, and 40 μg/mL X-gal. Then, the plate was cultured overnight at 37°C. The enzymatic signal was directly detected under white light, and the fluorescent signal was detected under UV light.
(TIF)
