# Supplementary figures and images for: Construction of a RFP-lacZα bicistronic reporter system and its application in lead biosensing
Source: PLoS One. 2020 Jan 30;15(1):e0228456. doi: 10.1371/journal.pone.0228456 (PMC6991944; doi:10.1371/journal.pone.0228456)

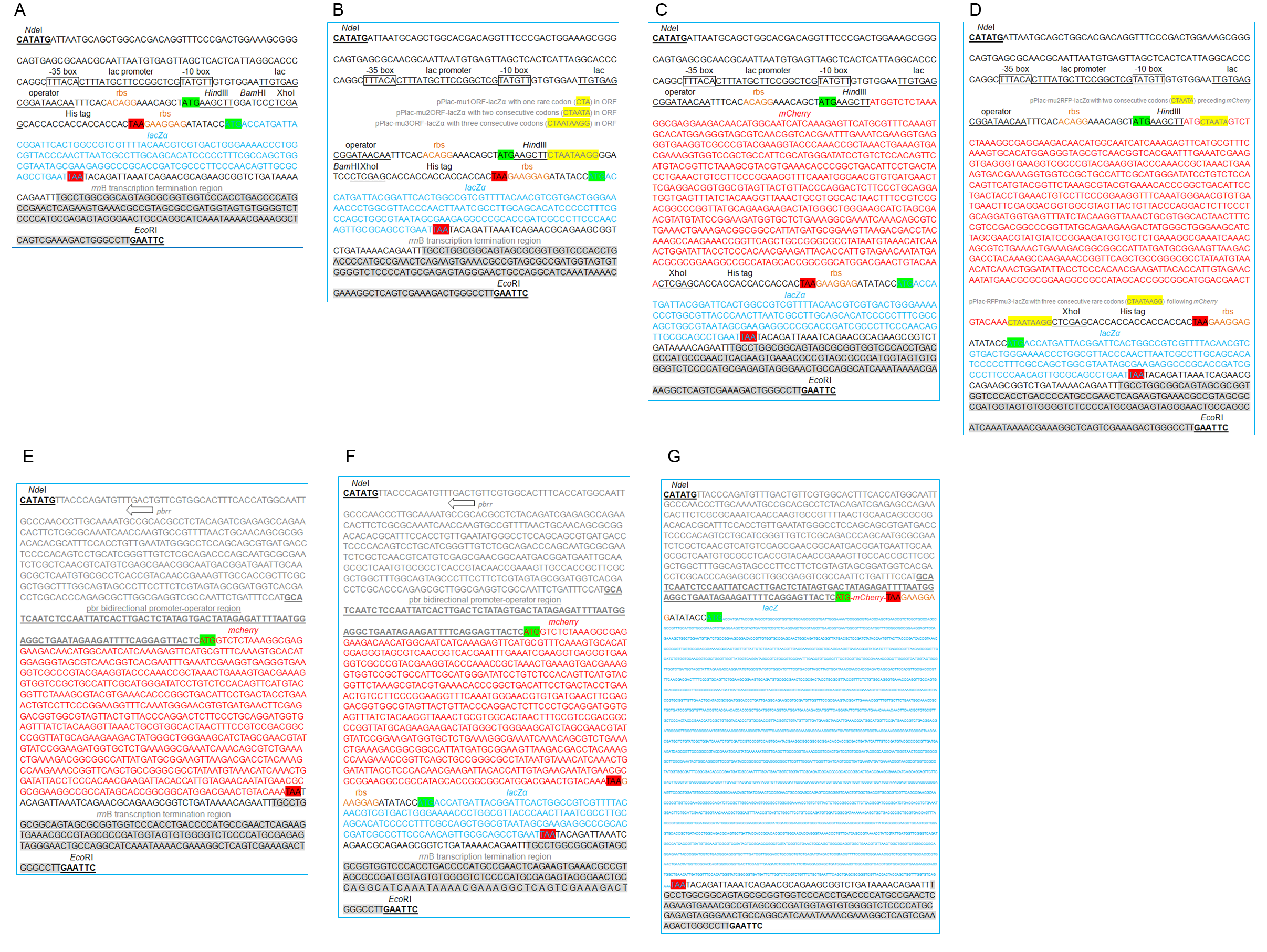

Supplement: S1 Fig — (A) The cloning/expression region of pPlac-ORF-lacZα. Another open reading frame (ORF) containing multiple cloning sites (MCS) is inserted immediately preceding the expression region of lacZα. (B) The cloning/expression region of pPlac-mu1ORF-lacZα, pPlac-mu2ORF-lacZα, and pPlac-mu3ORF-lacZα. One rare codon, two consecutive codons, or three consecutive codons were introduced into the first ORF in each pPlac-ORF-lacZα, respectively. (C) The cloning/expression region of the dicistronic expression vector pPlac-RFP-lacZα. The mCherry coding sequence was inserted as a HindIII/Xhol fragment into pPlac-ORF-lacZα. (D) The cloning/expression region of pPlac-mu2RFP-lacZα and pPlac-RFPmu3-lacZα. Two consecutive rare codons were inserted in front of mCherry to generate pPlac-mu2RFP-lacZα, and three consecutive rare codons were inserted behind mCherry to generate pPlac-RFPmu3-lacZα. (E) The cloning/expression region of pPpbr-RFP. The cassette including the pbrR gene and the divergent pbr promoter was inserted in front of mCherry. (F) The cloning/expression region of pPpbr-RFP-lacZα. The cassette including the pbrR gene and the divergent pbr promoter was inserted in front of the dicistronic mCherry-lacZα genetic element. (G) The cloning/expression region of pPpbr-RFP-lacZ. The cassette including the pbrR gene and the divergent pbr promoter was inserted in front of the dicistronic mCherry-lacZ genetic element. (TIFF) [file pone.0228456.s002.tiff]

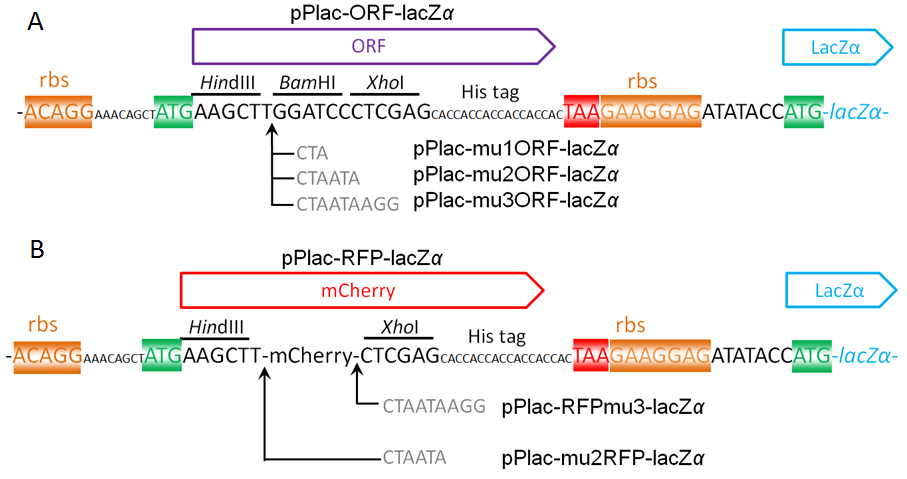

Supplement: S2 Fig — (A) The structure and DNA sequence of the ORF-lacZα reporter cassette in pPlac-ORF-lacZα. One, two, and three consecutive rare codons were introduced into the upstream ORF of pPlac-ORF-lacα after the HindIII site, generating pPlac-mu1ORF-lacZα, pPlac-mu2ORF-lacZα, and pPlac-mu3ORF-lacZα, respectively. (B) The structure and DNA sequence of the dual mCherry-lacZα reporter cassette in pPlac-RFP-lacZα, pPlac-mu2RFP-lacZα (containing two consecutive rare codons preceding mCherry gene), and pPlac-RFPmu3-lacZα (containing three consecutive rare codons following mCherry gene). (TIF) [file pone.0228456.s003.tif]

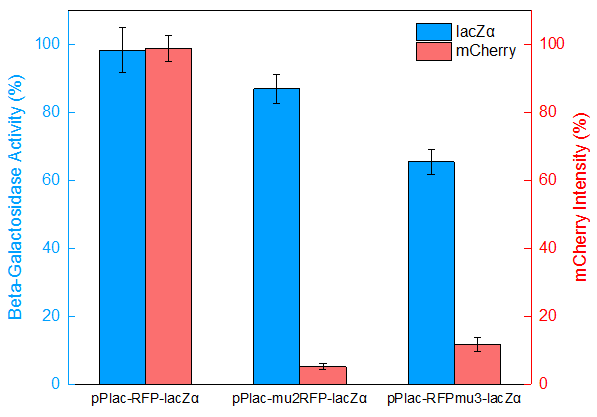

Supplement: S3 Fig — The mCherry fluorescence and β-galactosidase activity are shown as a percentage of the corresponding values in Top10/pPlac-RFP-lacZα, which are defined as 100%. The values are expressed as mean ± SD, n = 3. (TIF) [file pone.0228456.s004.tif]

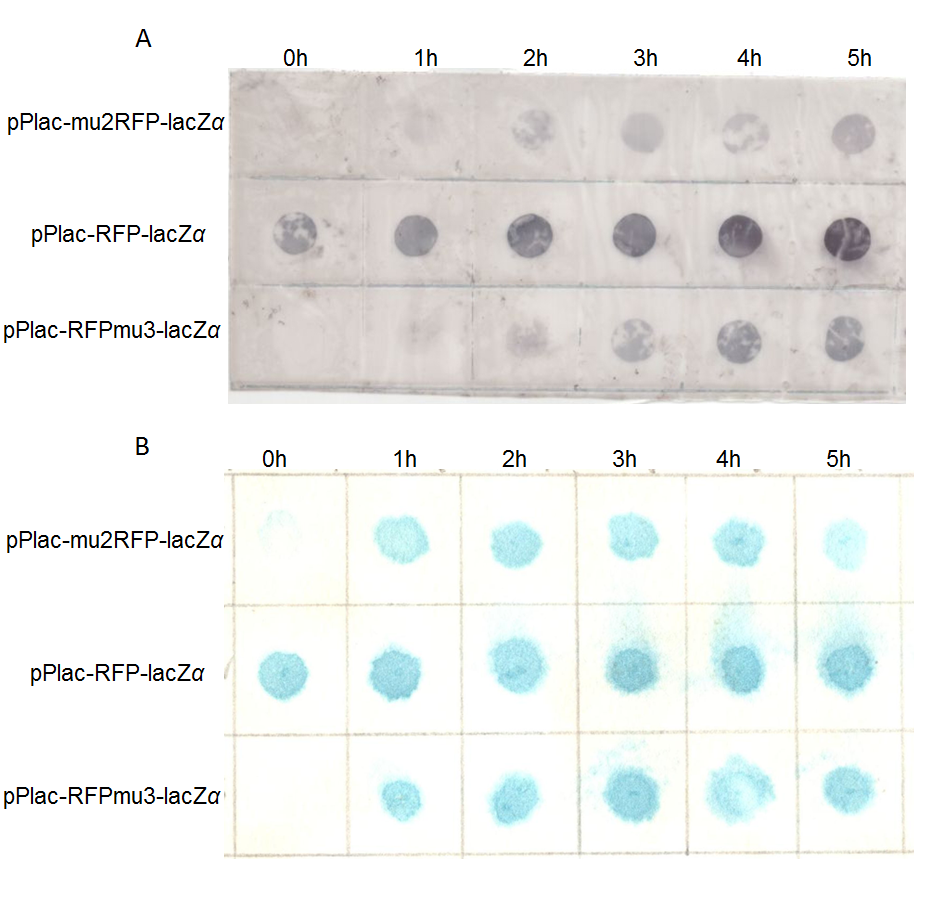

Supplement: S4 Fig — To assess mCherry production and β-galactosidase activity semi-quantitatively, a dot blot assay (A) was done to detect the His tag fused to the C-terminus of recombinant mCherry at different time intervals. A fast blot paper-based chromogenic method (B) was set up, and X-gal was chosen to be the substrate. (TIF) [file pone.0228456.s005.tif]

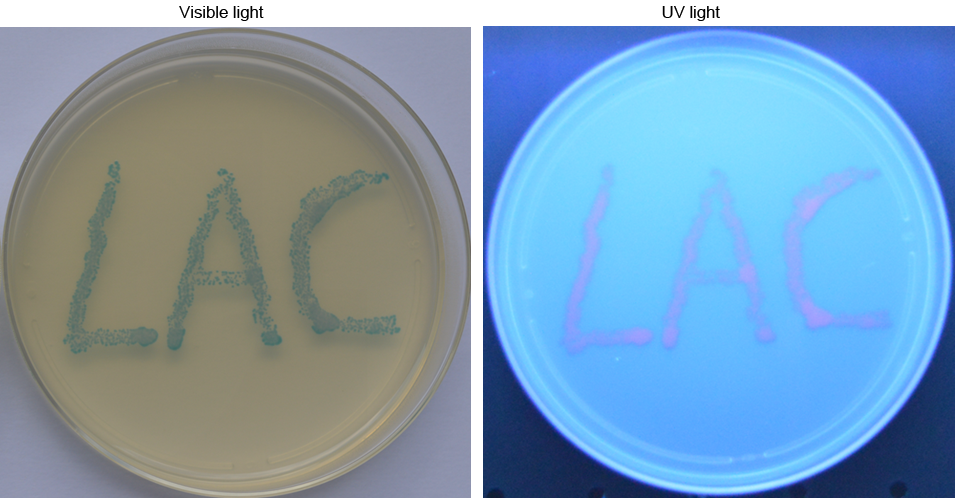

Supplement: S5 Fig — Recombinant Top10/pPpbr-RFP-lacZα was streaked on a LB agar plate containing 20 μM Pb(II), 0.1 mM IPTG, and 40 μg/mL X-gal. Then, the plate was cultured overnight at 37°C. The enzymatic signal was directly detected under white light, and the fluorescent signal was detected under UV light. (TIF) [file pone.0228456.s006.tif]
